# From Banff 1991 to Today: The Changing Landscape of the v-Lesion in Kidney Transplant Rejection

**DOI:** 10.3389/ti.2025.14818

**Published:** 2025-06-30

**Authors:** Karolien Wellekens, Priyanka Koshy, Candice Roufosse, Maarten Naesens

**Affiliations:** ^1^ Nephrology and Renal Transplantation Research Group, Department of Microbiology, Immunology and Transplantation, KU Leuven, Leuven, Belgium; ^2^ Department of Nephrology and Renal Transplantation, University Hospitals Leuven, Leuven, Belgium; ^3^ Department of Pathology, University Hospitals Leuven, Leuven, Belgium; ^4^ Department of Immunology and Inflammation, Imperial College, London, United Kingdom

**Keywords:** rejection, kidney transplant, TCMR, Banff classification, AMR

## Abstract

Intimal arteritis (v-lesion) has long been considered a hallmark of higher-grade T cell-mediated rejection (TCMR) in kidney transplantation, historically associated with poor graft survival and resistance to therapy. These associations have informed treatment strategies, often prompting intensified immunosuppression, including anti-thymocyte globulins (ATG). However, emerging evidence challenges the assumption that all v-lesions signify TCMR—particularly when they occur in isolation, without significant tubulo-interstitial inflammation. Recent observational studies and molecular analyses suggest that isolated v-lesions may instead reflect non-immune injury mechanisms, such as ischemia-reperfusion injury, particularly in the early post-transplant period. In addition, the shared nature of the v-lesion between TCMR and antibody-mediated rejection (AMR) raises concerns about overdiagnosis and potential overtreatment of “mixed rejection” phenotypes. Following advances in modern immunosuppression and improved donor-recipient matching, the clinical course of v-lesions may have evolved, with severe v3 presentations now rare—rendering historical comparisons less applicable to current practice. These insights highlight the need to revisit traditional paradigms and adopt a more nuanced, context-aware interpretation of v-lesions. This review integrates historical and contemporary perspectives, advocating for a reappraisal of the role of the v-lesion in kidney transplant biopsy evaluation.

## Introduction

Despite ongoing advances in transplant medicine, nearly 40% of kidney allografts fail within 10 years [1–3], with rejection remaining the primary cause [4, 5]. Rejection diagnosis relies on histopathological evaluation guided by the Banff classification [6], which primarily distinguishes T cell-mediated rejection (TCMR) from antibody-mediated rejection (AMR). The severity of TCMR is determined by histological findings, with moderate to severe tubular (t2-3) and interstitial inflammation (i2-3) defining grade I, while the presence of intimal arteritis upgrades the diagnosis to grade IIa (v1), IIb (v2), or III (v3), regardless of the extent of tubulo-interstitial inflammation. Intimal arteritis (“v”) is defined by the presence of inflammatory cells in the subendothelial space of one or more arteries [7]. Banff guidelines recommend evaluating at least two arteries for adequate v-lesion scoring. Treatment strategies for TCMR vary, with many U.S. centers favoring corticosteroids for TCMR grade I and a combination of steroids and anti-thymocyte globulin (ATG) for grade II or higher [8, 9], whereas ATG use is less common in Europe [10]. These strategies are largely informed by historical studies associating v-lesions with poorer graft survival and reduced treatment response [11–15].

Recent observational cohort studies [16–19] and molecular biopsy-based analyses [20, 21] have challenged the clinical significance of v-lesions. Emerging evidence suggests that isolated v-lesions—v-lesions occurring without substantial tubulo-interstitial inflammation—may not reflect the severe rejection phenotype traditionally defined by the Banff classification. This review examines the evolving understanding of TCMR and discusses how recent insights could inform and improve future strategies for diagnosing and managing rejection.

## Review

### Historical Assumptions on Prognostic Significance of the v-Lesion

**FIGURE 1:**
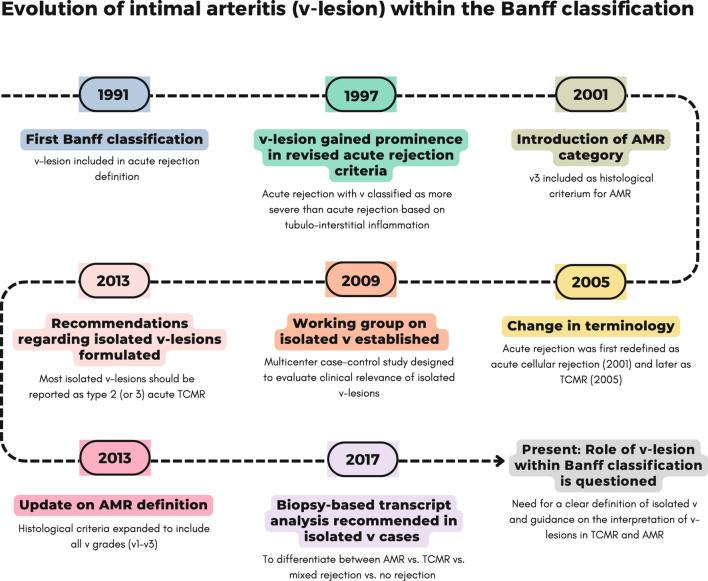
Evolution of intimal arteritis (v-lesion) within Banff classification across different updates. A detailed explanation of each update is provided in Table 1.

In the first Banff classification (1991) [22], the v-lesion was introduced as a defining feature of acute rejection. Mild-to-moderate intimal arteritis in at least one artery (v1) was classified as acute rejection grade II, while moderate-to-severe arteritis affecting more than one artery (v2) or severe intimal arteritis involving multiple arterial cross-sections and/or presenting with transmural arteritis, fibrinoid change, and smooth muscle necrosis (v3) was designated as grade III acute rejection. In this 1991 classification, significant interstitial inflammation (t2-3) with moderate tubulitis (t2) was designated as grade I acute rejection, while severe tubulitis (t3) also counted for grade II acute rejection (Table 1; Figure 1).

**TABLE 1:** Detailed explanation of Banff updates on intimal arteritis (v-lesion).

TCMR			
Year	Change	Evidence supporting change	Update in definitions (changes are underlined)
1991	First Banff classification established	Meeting report by Solez et al. [22]	Definition of acute rejection: Grade I: significant interstitial inflammation (>25% of parenchyma, i2-3) with moderate tubulitis (>4 mononuclear cells/tubular cross section or group of 10 tubular cells, t2) Grade II: significant interstitial inflammation (>25% of parenchyma, i2-3) with severe tubulitis (>10 mononuclear cells/tubular cross section, t3) and/or mild-moderate intimal arteritis in at least one artery (v1) Grade III: moderate-severe (v2) or severe (v3) intimal arteritis in multiple arteries and/or with transmural arteritis, fibrinoid change and smooth muscle necrosis
1997	v-lesion gained more prominence in acute rejection criteria	Colvin et al. [12] (1997) and Nickeleit et al. [13] demonstrated that intimal arteritis was associated with worse therapy response and reduced graft survival	Refinement of acute rejection definition, emphasizing the inferior outcome associated with intimal arteritis: Grade IA: significant interstitial inflammation (>25% of parenchyma, i2-3) with moderate tubulitis (>4 mononuclear cells/tubular cross section or group of 10 tubular cells, t2) Grade IB: significant interstitial inflammation (>25% of parenchyma, i2-3) with severe tubulitis (>10 mononuclear cells/tubular cross section, t3) Grade IIA: mild-moderate intimal arteritis (v1) Grade IIB: severe intimal arteritis comprising >25% of the luminal area (v2) Grade III: transmural arteritis and/or arterial fibrinoid change and smooth muscle necrosis with accompanying lymphocytic inflammation (v3) Additional change: v-grading was revised to be based on the most severely affected vessel due to the potential for sampling error in defining vasculitis
2005	Acute (cellular) rejection redefined as TCMR	The introduction of AMR in 2001 distinguished acute cellular from antibody-mediated rejection. Studies presented at Banff 2003–2005 meetings identified tubulitis as the primary correlate of T-cell-mediated effects [23–25]	No changes to grading
2009	Working group on isolated v was established	Formed in response to increasing uncertainty about the clinical relevance of isolated arteritis [26, 27]	A multicenter retrospective case-control study was initiated
2013	Recommendations regarding isolated v-lesions were formulated	The study [28] demonstrated that isolated v-lesions had comparable treatment response and graft survival to cases with v-lesions and significant tubulo-interstitial inflammation	“Most isolated v-lesions should be reported as type 2 (or 3) acute TCMR.”
Present	Role of v-lesion within Banff classification is challenged	Emerging evidence suggests isolated v may represent a non-rejection phenotype rather than TCMR Grade II–III [16–18, 20, 26, 29]	A potential change remains to be discussed
AMR			
2001	Introduction of AMR category	Studies presented at the Banff 2001 congress highlighted the critical role of antibodies in rejection, showing that biopsies with C4d deposition had significantly lower graft survival [25]	Three criteria must be present for AMR diagnosis: 1. Histologic evidence of acute tissue injury - ATN-like minimal inflammation - Capillary and or glomerular inflammation (ptc/g > 0) and/or thromboses - Arterial—v3 2. Evidence of antibody interaction with vascular endothelium 3. Serological evidence of donor-specific antibodies
2013	AMR definition expanded to include all v grades (v1–v3)	Lefaucheur et al. [30] demonstrated that AMR with v was associated with a worse prognosis than AMR without v.	Three criteria must be present for AMR diagnosis: 1.Histologic evidence of acute tissue injury • g > 0 and/or ptc>0 • v>0 • Acute thrombotic microangiopathy • Acute tubular injury 2. Evidence of antibody interaction with vascular endothelium 3. Serologic evidence of donor-specific antibodies
2017	Biopsy-based transcript analysis recommended in isolated v cases	Introduced in response to recurring diagnostic uncertainty in clinical practice [31]	Isolated v is a recommended indication for the use of molecular diagnostics in renal allograft biopsy interpretation to differentiate between AMR versus TCMR versus mixed rejection versus no rejection in cases of isolated v without MVI or TCMR, C4d-negative, with or without DSA.

At the Banff 1995 meeting [32], it was proposed to differentiate grade II acute rejection based on tubulitis versus arteritis, as these forms were suggested to have distinct pathogenesis and prognostic implications. It was demonstrated [12, 13] that intimal arteritis was associated with poorer graft survival and reduced response to therapy. This refinement was formally implemented in the 1997 classification [33], where grade II and higher were reserved for cases with intimal arteritis.

Since then, the v-lesion was considered a key marker of severe rejection, with studies from the 1990s and early 2000s consistently demonstrating its negative prognostic impact on kidney allograft survival. Higher v grades were associated with increased rejection severity and worse graft outcomes (Table 2). Kooijmans et al. [11] compared 42 biopsies with acute tubulointerstitial rejection, 18 with acute vascular rejection, and 7 with diffuse thrombosis, reporting that vascular rejection was associated with more frequent rejection episodes and higher allograft loss rates. Colvin et al. [12] found that vascular rejection (N = 24) resulted in worse clinical outcomes than rejection characterized by tubulitis with interstitial infiltration (N = 94). Nickeleit et al. [13] observed that patients with vascular rejection (N = 35) had a significantly lower response to steroid treatment compared to those with tubulitis and interstitial inflammation (N = 20), though both groups responded similarly to ATG and had comparable one-year graft failure rates.

**TABLE 2:** Key studies on intimal arteritis (1996–2025).

Study	Phenotypic study definitions	Study type	Sample size	Main findings
Kooijmans et al. [11]	Acute tubulointerstitial rejection: tubulitis with focal or widespread mononuclear cell infiltrates in the interstitium Acute vascular rejection: endothelial proliferation/swelling with intimal edema and/or mononuclear cell infiltration and adherence of mononuclear cells to the endothelium Diffuse thrombosis: necrosis of small arteries/arterioles, thrombosis in hilar/arcuate arteries, and fibrin thrombi in glomerular/peritubular capillaries	Observational cohort	N = 42 acute tubulointerstitial rejection, N = 18 acute vascular rejection, N = 7 diffuse thrombosis	Acute vascular rejection and diffuse thrombosis were associated with more frequent rejection episodes and higher allograft loss rates than acute tubulointerstitial rejection
Colvin et al. [12]	Type I rejection: mononuclear infiltrate in ≥5% of the cortex, tubulitis in ≥3 tubules and ≥2 of the following: edema, activated lymphocytes, or tubular injury Type II rejection: arterial, or arteriolar, endothelialitis, with or without type I features	Observational cohort	N = 94 type I, N = 24 type II	Type II rejection was clinically more severe than type I (peak serum creatinine >120% of baseline within 14 days after onset or incomplete response to antirejection therapy)
Nickeleit et al. [13]	Type I rejection: mononuclear infiltrate in ≥5% of the cortex, tubulitis in ≥3 tubules and ≥2 of the following: edema, activated lymphocytes, or tubular injury Type II rejection: endarteritis (mononuclear cells in the subendothelial space), with or without type I features. Type III rejection: fibrinoid arterial necrosis or transmural inflammation	Observational cohort	N = 20 type I, N = 35 type II, N = 4 type III	Type II rejection had poorer steroid response compared to type I, but responded equally to ATG. Type II had similar one-year graft survival and renal function at 6 and 12 months post- biopsy as type I. Type III did not respond to therapy, with all cases leading to graft failure within 12 months
Mueller et al. [14]	Borderline TCMR: mild tubulitis (t1) with mild interstitial infiltrate (i1) TCMR grade I: interstitial infiltrate (i2–3) and tubulitis (t2–3) TCMR grade I: intimal arteritis (v1–2) TCMR grade I: severe intimal arteritis (v3)	Observational cohort	N = 7 borderline TCMR, N = 11 TCMR grade I, N = 10 TCMR grade II, N = 7 TCMR grade III	Acute rejection severity correlated with treatment unresponsiveness and increased graft failure risk within 18 months post-biopsy. Intimal arteritis was the only histomorphologic predictor of poor survival. TCMR grade I–III groups had significantly higher serum creatinine at 1 and 2 years post-transplantation than matched controls
Minervini et al. [15]	t1: maximum tubulitis score of t1 t2: maximum tubulitis score of t2 t3: t3 tubulitis, characterized by the presence of >10 mononuclear cells/tubular cross-section or ≥2 areas of basement membrane destruction with i2/i3 inflammation and t2 tubulitis elsewhere in the biopsy v1: v1 intimal arteritis, irrespective of tubulitis grade v2: v2 intimal arteritis, irrespective of tubulitis grade v3: v3 intimal arteritis, irrespective of tubulitis grade	Observational cohort	N = 36 t1, N = 36 t2, N = 36 t3, N = 36 v, N = 18 v2, N = 11 v3	Severe tubulitis (t3) was associated with worse graft outcomes than mild/moderate tubulitis (t1/t2), approaching the outcomes of v1 intimal arteritis. Intimal arteritis (v2/v3) had the poorest prognosis, exceeding that of v1 arteritis and all tubulitis grades without coexisting arteritis
Haas et al. [34]	Grade IIA: mild to moderate intimal arteritis (1) Grade IIB: severe intimal arteritis (v2)	Observational cohort	N = 102 grade IIA, N = 29 grade IIB	Grade IIB cases had worse responses to therapy and higher rates of graft failure compared to grade IIA. However, grade IIB cases a had higher g + i + t score than grade IIA. Therapy response was associated with the extent of tubulitis and the composite g + i + t score (graft failure was not specifically analyzed in relation to g + i + t)
Mueller et al. [27]	Intimal arteritis with minimal tubulo-interstitial changes: v > 0, t < 2, i < 2	Transcriptomic profiling using microarray analysis	N = 143 biopsies +51 validation samples, *exact number of intimal arteritis with minimal tubulo-interstitial changes not explicitly stated*	Biopsies with intimal arteritis and minimal tubulointerstitial changes exhibited low expression of pathogenesis-based transcript sets
Kozakowski et al. [35]	Intimal arteritis: v > 0	Immunohistochemical staining of immune cells	N = 34 biopsies with intimal arteritis	Biopsies of intimal arteritis consist of a mixed infiltrate of monocytes/macrophages and T-lymphocytes, with a median CD68/CD3 ratio of 1.03. There was no correlation between the cellular composition of arterial and interstitial infiltrates and the proportion of interstitial and arterial macrophages did not impact graft survival
Sellarés et al. [26]	v0: v-lesion negative v ≥ 1: v-lesion positive (v1-v2-v3) Early: ≤1 year post-transplantation Late: >1 year post-transplantation	Observational cohort	N = 88 v0, N = 13 v1, N = 5 v2, N = 1 v3. Number of early and late v-positive biopsies not reported	v-lesions were not associated with allograft loss in early or late biopsies
Sun et al. [36]	Intimal arteritis: v > 0	Immunohistochemical staining of immune cells	N = 5 allografts resected because of irreversible graft failure, all classified as AMR with intimal arteritis	Macrophages and T-lymphocytes were the predominant immune cells, with CD8^+^ cytotoxic T-cells comprising 45.4% of T-cells. Neutrophils and NK cells were also present, with a higher proportion of neutrophils in v3 vasculitis compared to v2 lesions
Reeve et al. [37]	Isolated v: v > 0 and (i < 2 and/or t < 2)	Transcriptomic profiling using microarray analysis	N = 403 biopsies, including N = 24 with isolated v	19 out of 24 biopsies (79%) with isolated v-lesions had low TCMR scores (≤0.1)
Halloran et al. [38]	Isolated v: i < 2 or t < 2 and v > 0	Transcriptomic profiling using microarray analysis	N = 300 biopsies, including N = 35 TCMR and N = 22 TCMR + AMR histological diagnoses	Among 35 TCMR cases, 11 were molecular score-negative/histology-positive, and among 22 TCMR + AMR cases, 5 were molecular score-negative/histology-positive. Of these, 2/11 TCMR and 1/5 TCMR + AMR discrepancies were attributed to TCMR diagnoses based on the isolated v-lesion criterion
Lefaucheur et al. [30]	TCMR/v-, TCMR/v+, AMR/v-, AMR/v+: classification based on hierarchical cluster and principal component analysis. Groups were defined using combined histological lesions, C4d and DSA status	Observational cohort	N = 139 TCMR/v-, N = 26 TCMR/v+, N = 73 AMR/v-, N = 64 AMR/v+	Risk of graft loss was similar between TCMR/v- and TCMR/v + groups. AMR/v+ was associated with worse graft survival compared to AMR/v-
Sis et al. [28]	Isolated v: v > 0, t ≤ 1, i ≤ 1 TCMR grade I with v: v > 0, t2-3i2-3 Negative controls: v0, t0-1i0-1	Observational cohort	N = 103 isolated v, N = 101 TCMR grade I with v and N = 103 negative controls	Isolated v had similar treatment response rates and graft survival to TCMR grade I with v
Salazar et al. [20]	Isolated v: v > 0 and (i < 2 or i < 2), with/without AMR (as defined by Banff 2013 criteria) i-t-v lesions: v > 0 and (i ≥ 2 and t ≥ 2), with/without AMR Early: ≤1 year post-transplantation Late: ≤1 year post-transplantation Negative controls: v0	Observational cohort + transcriptomic profiling using microarray analysis	N = 28 isolated v, N = 21 i-t-v lesions, N = 654 v-negative. AMR in 17/49 v-positive biopsies. Early biopsy in 28/49, late in 21/49 v-positive biopsies	v-lesions did not increase the risk of graft failure compared to biopsies without v-lesions, regardless of the presence of concomitant AMR. Molecular TCMR scores were positive in 95% of i-t-v lesions and 21% of isolated v-lesions. Among 12 early isolated v-lesion biopsies, 9 showed no molecular rejection. Between 1 and 5 years, 4/5 had molecular rejection (1 TCMR, 1 AMR, 2 TCMR + AMR). Beyond 5 years, 9/11 had positive AMR scores, but only 1 had a positive TCMR score
Wu et al. [39]	sAMRV: suspicious AMR with v AMRV: AMR with v TMRV: TCMR with v All categories were defined according to the Banff 2013 criteria	Observational cohort	N = 37 sAMRV, N = 33 AMRV and N = 78 TCMRV. N = 80 v1, N = 51 v2 and N = 17 v3 cases (in total)	TCMRV, sAMRV, and AMRV showed similar responses to antirejection therapy, whereas v2 and v3 lesions were associated with significantly poorer outcomes than v1. Eight-year death-censored graft survival was higher in TCMRV than in AMRV, and significantly better in v1 compared to v2 or v3
Rabant et al. [16]	Isolated v: v1-3, i ≤ 1 with any t-score, g + ptc≤1 and C4d negative AMR with v: AMR criteria present with v-lesion	Observational cohort	N = 33 isolated v N = 54 AMR with v	Graft survival in AMR with v was significantly lower than in isolated v
Novotny et al. [17]	Isolated v: v1-3, t < 2, i < 2, g + ptc = 0, C4d0, DSA negative TCMR with v: v1-3, t0-3, i0-3, g + ptc = 0, C4d0, DSA negative Suspected AMR with v: v1-3, t0-3, i0-3, g + ptc 0-6, C4d 0-3, DSA negative/positive AMR with v: v1-3, t0-3, i0-3, g + ptc 0-6, C4d 0-3, DSA positive	Observational cohort	N = 25 isolated v, N = 18 TCMR with v, N = 36 suspected AMR with v and N = 19 AMR with v	Isolated v responded well to steroids, showed no persistence of arteritis in follow-up biopsies, and had favorable graft function and survival. AMR with v had significantly worse graft survival compared to isolated v
Mikhail et al. [18]	Isolated v1 i-t-v lesions *Criteria not explicitly stated*	Observational cohort	N = 50 v1, N = 28 i-t-v lesions	Isolated v1 had a better response to therapy and superior graft survival compared to i-t-v lesions
Novotny et al. [40]	AMR with v: v > 0 and presence of MVI ± C4d ± DSA AMR without v: presence of MVI ± C4d ± DSA	Observational cohort	N = 36 AMR with v, N = 102 AMR without v	AMR with v was a significant risk factor for the development of transplant glomerulopathy, regardless of DSA status, but was not associated with graft failure at 36 months
Nankivell et al [29]	Isolated VR: v > 0, i < 1, t < 1) Inflamed VR: v > 0, i ≥ 1, t ≥ 1 irrespective of C4d and DSA Early: ≤1 month post-transplantation Late: >1 month post-transplantation	Observational cohort	N = 34 isolated VR, N = 66 inflamed VR; early VR in 51%, late VR in 49%	Isolated VR cases had better graft survival compared with inflamed VR. Early intimal arteritis was associated with delayed graft function and transient elevations in serum creatinine, but overall responded well to treatment. Late intimal arteritis was more frequently linked to chronic fibrosis and subsequent graft loss
Rosales et al. [41]	Intimal arteritis: v > 0	Transcriptomic profiling using NanoString B-HOT panel	N = 326 kidney transplant biopsies	Molecular TCMR scores correlated more strongly with t and i than with v
Sikosana et al. [42]	Intimal arteritis: v > 0	Transcriptomic profiling using microarray analysis	N = 1679 kidney transplant biopsies	Molecular TCMR scores were primarily associated with t and i, but not with v
Buxeda et al. [21]	Isolated endarteritis: v > 0, t0-1, i0-1, g + ptc 0-1, C4d negative Early: ≤1 month post-transplantation Late: >1 month post-transplantation AMR v+: v > 0 cases meeting 2019 Banff criteria for AMR, and those with isolated microvascular inflammation ≥2 C4d-negative *TCMR v+ and mixed rejection v + criteria not explicitly stated*	Observational cohort + transcriptomic profiling using NanoString B-HOT panel	N = 10 early isolated v, N = 13 late isolated v, N = 26 AMR v+, N = 10 TCMR v+, N = 23 mixed rejection v+	Early isolated v-lesions had higher delayed graft function rates and the worst 1-year death-censored graft survival compared to late isolated v-lesions and other rejection subtypes. Early isolated v showed increased early/acute injury gene expression but lower activation of rejection-related pathways, with reduced TCMR and AMR gene expression compared to TCMR, AMR, and mixed rejection
Wellekens et al. [19]	Isolated v: v > 0, t0-3i0 or t0i0-3, not meeting (p)AMR-MVI criteria) Borderline changes with v: v > 0, t1-3i1 or t1i1-3, not meeting (p)AMR-MVI criteria TCMR grade I with v: v > 0, t2-3i2-3, not meeting (p)AMR-MVI criteria (p)AMR-MVI with v: v > 0, t0-3i0-1 or t0-1i0-3, meeting Banff 2022 criteria3 for (probable) AMR or DSA-negative C4d-negative MVI TCMR-I + (p)AMR-MVI with v: v > 0, t2-3i2-3, meeting (p)AMR-MVI criteria	Observational cohort	N = 166 isolated v, N = 87 borderline changes with v, N = 66 TCMR grade I with v, N = 148 (p)AMR-MVI with v and N = 67 (12.5%) TCMR-I + (p)AMR-MVI with v	Although borderline changes with v showed slightly higher 10-year graft failure rates than isolated v-lesions, cases of TCMR-I, (p)AMR-MVI and TCMR-I + (p)AMR-MVI with v were associated with significantly worse outcomes. In a matched analysis of 534 v-positive cases and v-negative controls, v-lesions had no significant impact on graft outcomes

Further supporting these findings, Mueller et al. [14] analyzed 35 biopsies (7 borderline, 11 TCMR grade I, 10 TCMR grade II, 7 TCMR grade III), demonstrating that TCMR grade I-III correlated with treatment unresponsiveness and increased graft failure risk. Minervini et al. (2000) [15] compared 36 cases each of t1, t2, and t3 tubulitis, as well as 36 v1, 18 v2, and 11 v3 cases, irrespective of t scores. These findings confirmed that v2/v3 intimal arteritis had the poorest prognosis, surpassing v1 and all tubulitis grades without concurrent intimal arteritis, while severe tubulitis (t3) had outcomes comparable to v1.

Several limitations of earlier studies warrant consideration. Many did not account for concomitant tubulointerstitial or microvascular inflammation—both of which can independently affect outcomes—and often failed to assess donor-specific antibodies (DSA). Inclusion criteria for acute rejection subtypes were inconsistent, with some studies not adhering to Banff-defined thresholds. Most lacked multivariable analyses to adjust for confounding clinical factors, were limited by small sample sizes, and frequently included repeat biopsies, complicating survival analysis by counting some patients multiple times. Additionally, follow-up durations were often insufficient to capture the long-term impact of rejection phenotypes.

### The Concept of “Isolated v” as a Distinct Phenotype

At the Banff 2009 conference [43], a working group on isolated v was established to assess its significance in renal allograft biopsies. This initiative stemmed from growing uncertainty regarding the clinical relevance of isolated arteritis in biopsies that lacked significant tubulointerstitial inflammation and, therefore did not meet the Banff criteria for grade I TCMR. A study by Sellarès et al. [26] found no correlation between v-lesions and graft failure risk, whether analyzed as v0 (N = 88) versus v > 0 (N = 19) or stratified by intimal arteritis severity (v1-2-3). Additionally, microarray analyses [27] revealed minimal inflammatory gene expression, including low levels of T cell activation, IFN-γ, and chemokines, in isolated v biopsies.

These findings led to the initiation of a multicenter retrospective case-control study to further investigate the clinical implications of isolated v. Presented at the 2013 Banff conference [44], the study defined isolated v as intimal arteritis with minimal interstitial inflammation (i ≤ 1) and tubulitis (t ≤ 1) [28]. The results demonstrated that isolated v (N = 103) had comparable treatment response rates and graft survival to v-lesions with significant tubulo-interstitial inflammation (N = 101, v1 with t2-3, i2-3). The Banff 2013 meeting report suggested that “*most isolated v-lesions should be reported as type 2 (or 3) acute TCMR.”* [44]

However, several limitations should be acknowledged. Some patients had concurrent AMR or microvascular inflammation (MVI), likely confounding the findings. As a case-control study, it was also inherently prone to selection bias. Importantly, the study did not address cases with v1 in combination with either t2–3/i0–1 or t0–1/i2–3, leaving uncertainty about how to classify and manage such presentations. Moreover, the conclusion that “most” isolated v-lesions represent severe TCMR remains vague, as no practical guidance was offered to support its implementation in clinical decision-making.

### Recent Data on the Clinical Relevance of Isolated v-Lesions

Over the past decade, accumulating evidence suggests that isolated v may represent a benign phenotype, with multiple studies reporting favorable outcomes. Isolated v has been associated with treatment responses and graft survival rates comparable to—or even better than—those observed in TCMR with significant tubulo-interstitial inflammation (Table 2). Salazar et al. [20] found that the presence of v-lesions (N = 49) did not increase the risk of graft failure compared to v-negative biopsies (N = 654). Novotny et al. [17] concluded that isolated v cases (N = 25) responded well to steroid treatment, showed no persistence of arteritis in follow-up biopsies, and had favorable graft outcomes. Similarly, Nankivell et al. [29] demonstrated that isolated v (N = 34) was associated with better graft survival than vascular rejection cases with concomitant tubulo-interstitial inflammation (N = 66). Also, Lefaucheur et al. [30] found no significant difference in outcomes up to 72 months post-transplant when comparing TCMR with v-lesions (N = 26) to TCMR without v-lesions (N = 139). In a multicenter observational cohort study [19] (707 v-lesion positive biopsies out of a total of 16,774 biopsies, corresponding to 534 transplants) we reported that isolated v-lesions were linked to superior 10-year graft survival compared to biopsies with t and i scores meeting or exceeding the borderline threshold, as well as biopsies fulfilling (probable) AMR/MVI criteria in the absence of significant tubulo-interstitial inflammation. Furthermore, when matching the 534 v-positive cases to v-negative controls, no significant impact of v-lesions on outcomes was observed.

A 2015 study [39] comparing 80 v1 cases to 51 v2 and 17 v3 cases concluded that higher-grade v-lesions (v2 and v3) were associated with significantly poorer response to rejection treatment and lower 8-year death-censored graft survival, with outcomes being more favorable in v1 cases. The study included patients who received kidney transplants between 1996 and 2012. It should be noted that the prevalence of v-lesions in recent observational cohort studies was relatively low, reported at, 2.7% [29], 4% [19, 30], 7% [20] and 9.7% [17], with the majority classified as mild (v1). The rarity of v3 lesions has generally limited the ability to perform meaningful analyses of this severe phenotype in recent studies.

Several factors likely contribute to the low detection rate of v-lesions in kidney allograft biopsies. Most biopsies contain only one to five arterial cross-sections, as arteries represent a small fraction of the total biopsy area compared to tubules and glomeruli. Moreover, rejection-related inflammation is often focal, and arteritis may affect only a segment of the arterial circumference, reflecting a patchy distribution. Together, these factors increase the risk of sampling bias. In addition, reproducibility may also be limited by interobserver variability, as some pathologists rely solely on morphology, while others incorporate immunohistochemical staining.

Nonetheless, the vascular rejection phenotype described in earlier studies may not reflect what is commonly observed today. Historically, vascular rejection was often hyperacute, characterized by severe arteritis, thrombosis, and frequent graft loss. Advances in immunosuppressive therapies [45, 46] and improved donor-recipient matching—particularly the avoidance of recipients with pre-transplant donor-specific HLA antibodies [47]—have significantly reduced the incidence of such severe presentations. Moreover, the current Banff definition of intimal arteritis has a notably low diagnostic threshold, requiring only a single inflammatory cell in the subendothelial space to classify a biopsy as v1 (TCMR grade IIA) [7] This raises the question of whether the v-lesions reported in older studies represent the same pathological process examined in more recent research.

### Clinical Factors Related to Isolated v-Lesions

Timing post-transplantation is a key factor in the interpretation of isolated v-lesions. Rabant et al. [16] (N = 33) reported a median of 27 days post-transplantation for biopsies demonstrating isolated v-lesions. Nankivell et al. [29] found that early intimal arteritis (≤1 month, N = 51) was associated with delayed graft function (DGF) and transient elevations in serum creatinine, but generally responded well to therapy. In contrast, late-onset arteritis (>1 month, N = 49) was more frequently associated with chronic fibrosis and graft loss. Buxeda et al. [21] reported that early (≤1 month) isolated v-lesions (N = 10) were associated with the poorest 1-year death-censored graft survival when compared to late isolated v-lesions (N = 13), or to TCMR with v (N = 10), AMR with v (N = 26), and mixed rejection with v (N = 23). In this cohort, 75% of early isolated v cases were associated with DGF, 60% progressed to primary non-function, and all graft losses in this group were due to this early event. However, the study lacked long-term follow-up, and the high proportion of expanded criteria donor kidneys likely increased vulnerability to delayed graft function [48–50]. In contrast, Sellarés et al. [26] (N = 19) compared the prognostic significance of intimal arteritis in biopsies performed within the first year (≤1 year) versus after 1 year (>1 year). Unlike studies focused on the immediate post-transplant period (e.g., ≤1 month), this analysis did not find an association between v-lesions and allograft loss.

In addition, early isolated v-lesions are frequently identified in the absence of donor-specific anti-HLA antibodies (DSA). Several studies [16, 19–21] have reported low DSA positivity rates in early isolated v-lesions, in contrast to higher DSA prevalence in late-onset v-lesions or in cases associated with AMR or mixed rejection.

These findings have led to the hypothesis that early isolated v-lesions may reflect non-immune vascular injury—such as vascular trauma or ischemia-reperfusion injury related to the transplantation procedure—rather than alloimmune rejection [16, 17, 20].

Finally, arteritis is not pathognomonic for rejection. Arterial inflammation may also result from recurrent or *de novo* vasculitis (e.g., ANCA-associated vasculitis, cryoglobulinemia, IgA vasculitis) or appear adjacent to vascular thrombosis, even in the absence of histological rejection.

### v-Lesions in the Context of AMR

While the definitions by the Banff Working Group of “isolated v” were developed in relation to full TCMR [28], another important aspect of intimal arteritis in kidney transplant biopsies, is its non-specificity for TCMR. When AMR was first defined in 2001, v3 was already included among the histological criteria (Table 1; Figure 1). At the 2013 Banff meeting [44], it was further concluded that v1 and v2 should also be considered part of the AMR histologic criteria. This decision was based on findings by Lefaucheur et al. [30], who reported that AMR with v was associated with a worse prognosis than AMR without v. However, only eight out of 64 HLA-DSA-positive cases in their study had v-lesions as the only histologic feature of AMR, making it impossible to assess outcomes specifically in this subgroup. Additionally, AMR with v was associated with a higher incidence of t- and i-lesions, which correlated with graft loss. Since the primary analysis comparing AMR with and without v did not adjust for concomitant tubulointerstitial inflammation, it remains unclear whether the worse outcomes in AMR with v cases were driven by the v-lesion itself or by coexisting tubulointerstitial inflammation.

Since then, the relationship between AMR and intimal arteritis, in the absence of TCMR, has been studied only scarcely (Table 2). Rabant et al. [16] found that graft survival up to eight years after the index biopsy was significantly lower in AMR with v (N = 54) compared to isolated v cases (N = 33). Similarly, Novotny et al. [17] reported that AMR with v (N = 19) was associated with worse three-year graft survival compared to isolated v (N = 25). In addition, the presence of intimal arteritis in AMR was a significant risk factor for progression to transplant glomerulopathy, irrespective of DSA status, but was not associated with an increased risk of graft failure at 36 months [40]. Salazar et al. [20] also concluded that the presence of v-lesions did not increase the risk of graft failure when analyzed in biopsies diagnosed with AMR (N = 17). In a recent multicenter observational cohort study (707 v-lesion positive biopsies, derived from 534 transplants) [19], we observed no difference in 10-year graft survival between cases with a Banff category 2 diagnosis and a v-lesion and those with a category 2 diagnosis without a v-lesion. The study also highlighted the risk of overinterpreting the presence of a v-lesion in (probable)AMR/MVI cases as indicative of mixed rejection when significant tubulo-interstitial inflammation is absent. Notably, cases diagnosed as “mixed rejection” solely due to the presence of a v-lesion had better clinical outcomes than those with both (probable)AMR/MVI and marked tubulo-interstitial inflammation—classified in the study as true “mixed rejection.”

Nevertheless, it is important to note that in studies using biopsies obtained before 2013, v1 and v2 lesions were not included in the diagnostic criteria for AMR. As a result, v-lesions in the context of AMR may have been interpreted and managed as TCMR, in accordance with the Banff classification at that time.

### The Pathogenesis of Intimal Arteritis

Since 2017, the Banff classification has recommended biopsy-based transcript analysis to support the differential diagnosis of AMR, TCMR, mixed rejection in isolated v cases that lack MVI and TCMR features, are C4d-negative, and may or may not have DSA [31]. This recommendation underscores the added value of molecular tools to clarify ambiguous cases.

Reeve et al. [37] developed a TCMR score using microarray analysis of 403 kidney transplant biopsies and found that while the score correlated strongly with interstitial inflammation (i) and tubulitis (t), 79% of isolated v-lesions had low TCMR scores. A prospective validation study [38] (N = 300) confirmed that discordance between histology and molecular findings often stemmed from TCMR diagnoses based solely on isolated v-lesions. In a third study, the same team [20] compared 28 isolated v-lesions to 21 biopsies with i-t-v lesions and found that 95% of the latter had positive TCMR scores, whereas only 21% of isolated v-lesions did. Most early (≤1 year post-transplantation) isolated v-lesions lacked molecular rejection signals, whereas those occurring beyond 1 year more frequently exhibited molecular AMR signatures. Logistic regression models trained on molecular diagnoses confirmed that TCMR scores were primarily driven by i and t, but not v [42]. Similarly, studies using the NanoString Banff Human Organ Transplant (B-HOT) panel [51] showed that TCMR-associated gene sets correlated strongly with i and t [41], but not with v [21]. Moreover, early isolated v-lesions (≤1 month post-transplant) demonstrated increased expression of early injury-response genes compared to late-onset cases (>1 month), further supporting a non-immune injury mechanism in the early post-transplant period. While advances in molecular diagnostics have enhanced our understanding of these biopsies, the focal nature of intimal arteritis may still lead to false-negative results due to sampling limitations [52].

Nevertheless, the underlying pathogenesis and cellular composition of intimal arteritis remain poorly characterized. While earlier studies primarily associated intimal arteritis with CD4^+^ and CD8^+^ T cell infiltration, more recent evidence suggests a broader immune response involving both T lymphocytes and macrophages [35, 36]. A recent study [53] using deconvolution of bulk transcriptomic data found that v-lesions were most strongly associated with innate immune cells—such as NK cells and monocytes/macrophages—similar to other vascular lesions like peritubular capillaritis and glomerulitis.

These findings suggest that innate immunity may contribute to the pathogenesis of intimal arteritis. In particular, early isolated v-lesions may reflect endothelial stress and innate immune activation following ischemia-reperfusion injury. This process induces endothelial and parenchymal damage, releasing damage-associated molecular patterns (DAMPs) [54–57] engage pattern recognition receptors (PRRs), such as Toll-like receptors (TLRs), on endothelial and immune cells. PRR signaling promotes cytokine release, interferon production, and regulated cell death, amplifying local inflammation and recruiting additional immune cells. Concomitantly, endothelial activation upregulates adhesion molecules (e.g., P-selectin, ICAM-1), promoting platelet adhesion and degranulation. Platelet-derived mediators amplify inflammation and recruit monocytes, neutrophils and T cells [58]. In parallel, stress-induced changes in HLA expression—such as downregulation of classical HLA-I and upregulation of non-classical HLA-I and NKG2D ligands (e.g., MICA, MICB, ULBPs)—can trigger NK cell–mediated cytotoxicity [59, 60]. Persistent injury may also expose cryptic self-antigens (e.g., AT1R, perlecan, collagen V), triggering non-HLA autoantibody responses [61, 62]. These antibodies can form immune complexes that activate Fc receptors or complement pathways, amplifying cytokine release, endothelial damage, and vascular inflammation. However, whether these mechanisms directly contribute to the pathogenesis of intimal arteritis remains uncertain and warrants further investigation.

Given their association with favorable outcomes, early isolated v-lesions are presumed to potentially resolve spontaneously if the inciting insult wanes and immune activation diminishes. However, it remains unclear whether and how this resolution occurs, and whether there is a place for specific therapeutic interventions.

The mechanisms underlying intimal arteritis in TCMR also remain incompletely understood. Donor HLA antigens activate recipient T cells via direct or indirect allorecognition [63], promoting T cell–endothelium interactions through upregulated HLA expression. This may lead to cytokine release and direct cytotoxicity via perforin/granzyme or Fas/FasL pathways [64, 65]. Alternatively, v-lesions—like peritubular capillaritis—may result from increased leukocyte trafficking in areas of interstitial inflammation, without direct endothelial targeting. In contrast, the mechanisms driving arteritis in AMR are better defined: DSAs bind activated endothelium, triggering complement activation and antibody-dependent cellular cytotoxicity (ADCC), primarily mediated by NK cells or monocytes via Fc receptor engagement [66]. In both TCMR and AMR, immune infiltration into the subendothelial space gives rise to intimal arteritis, which can progress to transmural inflammation, smooth muscle necrosis, fibrin deposition, and vascular occlusion in severe cases.

While much remains unknown, v-lesions are clearly a heterogeneous finding whose clinical significance depends on timing post-transplant, immunologic risk (e.g., HLA-DSA status), and coexisting features suggestive of AMR, TCMR, or recurrent disease. Traditional histology offers limited specificity in isolated v-lesions, but emerging tools—such as (spatial) transcriptomics, integrated within multi-omics frameworks—may offer new biological insights. Refining the diagnostic role of v-lesions in the Banff classification will require a context-aware approach that accounts for both immune and non-immune causes of vascular injury.

## Discussion

The Banff classification has provided a crucial framework for diagnosing rejection in kidney transplantation, enabling consistency in histopathological assessment across centers worldwide. However, as transplant medicine evolves, so too must our understanding of the clinical significance of specific histological lesions.

Long regarded as a hallmark of moderate-to-severe T cell-mediated rejection (TCMR), the v-lesion is now recognized as a heterogeneous entity. Emerging evidence challenges the assumption that all v-lesions signify TCMR, particularly when they occur in isolation without substantial tubulo-interstitial inflammation. Furthermore, advances in immunosuppressive therapy and improved donor-recipient matching may have fundamentally altered the clinical course of intimal arteritis, raising doubts about whether historical interpretations remain applicable to contemporary transplant populations.

A clear and consistent definition of “isolated v” is essential before its role within the Banff classification can be meaningfully reconsidered. The 2009 Banff isolated v working group [28, 43] proposed defining “isolated v” as intimal arteritis with minimal interstitial inflammation (i ≤ 1) and tubulitis (t ≤ 1). However, cases fulfilling the Banff criteria for borderline TCMR (t1i1), and having v lesions, should likely not be called isolated v, as this would lead to the contradiction that “borderline TCMR” is often treated [9, 10], while the term “isolated v” designates a potentially more benign phenotype. In recent study [19] we proposed a definition more aligned with current Banff thresholds, distinguishing isolated v (v > 0, t0–3i0 or t0i0–3) from borderline TCMR (v0, t1–3i1 or t1i1–3), TCMR I (v0, t2–3i2–3), and TCMR II–III (v > 0, t1–3i1–3). While classifying t2–3i0 or t0i2–3 as isolated v may seem counterintuitive, such cases were rare (2.8% of v-lesion positive biopsies) in our study. In these instances, case-level pathology review is essential to determine whether tubulitis or interstitial inflammation is truly isolated. Tools such as the activity indices by Vaulet et al. [67] or molecular diagnostics such as the B-HOT panel [51] may help refine classification and inform treatment decisions in these exceptional cases.

Second, the prior definitions used by the Banff Working Group [28] did not account for the relationship between intimal arteritis and AMR, leaving it unclear whether v-lesions in AMR represent concurrent TCMR (“mixed rejection”). This is further complicated by the lack of a clear Banff definition for mixed rejection, making shared lesions like v difficult to interpret and potentially leading to overdiagnosis and overtreatment of mixed phenotypes [19]. In such cases, the v-lesion may be more appropriately interpreted as a manifestation of AMR, particularly when other histologic features of TCMR are absent. A more coherent approach would classify these as (probable) AMR/MVI with v, and reserve “isolated v” for biopsies lacking both AMR/MVI and tubulo-interstitial criteria for (borderline) TCMR. Refining the role of the v-lesion within the Banff classification does not mean abandoning its significance but rather integrating new insights to enhance diagnostic precision. Importantly, clinical practice is already evolving, as a recent survey indicated that many European transplant centers no longer treat isolated v-lesions with ATG [10].

The Banff process has always been one of evolution, shaped by emerging data and expert consensus. The challenge now is how best to incorporate these new insights while maintaining a classification system that remains clinically relevant and widely applicable. Over a decade ago, Salazar et al. [20] cautioned that “isolated v-lesions have been seriously misinterpreted for over 20 years, potentially leading to inappropriate treatment decisions.” With accumulating clinical and molecular evidence, the Banff classification may need to adapt and reconsider the specific definition of an “isolated v” phenotype.
